# Effects of Soil Fumigant-Mediated Changes in the Microbial Communities of Soil with Continuous Cropping on Tomato Yield and Soil-Borne Diseases

**DOI:** 10.3390/microorganisms14020400

**Published:** 2026-02-07

**Authors:** Yan Li, Ran Wu, Songnan Jia, Fengcui Fan, Jingsong Li, Shengyao Liu

**Affiliations:** 1 Shijiazhuang Academy of Agricultural and Forestry Sciences, Shijiazhuang 050041, China; yoginlily@126.com (Y.L.); candy58906@163.com (R.W.); 2 Institute of Agriculture Information and Economics, Hebei Academy of Agriculture and Forestry Sciences, Shijiazhuang 050051, China; hbjiasongnan@126.com (S.J.); njsffc@163.com (F.F.); lijingsongsjz@163.com (J.L.)

**Keywords:** dazomet, metham sodium, calcium cyanamide, seedling recovery stage, fruiting stage, bacteria, fungi

## Abstract

To scientifically assess the effects of environmentally friendly fumigants on soil microbial communities, soils from a 7-year continuous cropping tomato greenhouse were studied, with unfumigated soil used as the control (CK). Rhizosphere soil samples treated with dazomet (DZ), metam sodium (MS) and calcium cyanamide (CC) were collected at the seedling recovery and fruiting stages. The influences of different fumigants and growth stages on soil microbial communities, tomato yield and soil-borne diseases were investigated. The results indicated that soil fumigation significantly decreased microbial community richness and diversity at the seedling recovery stage, which gradually recovered at the fruiting stage. The variation trends of microbial relative abundance at the phylum and genus levels differed among the treatments at both stages. At the phylum level, Actinobacteria and Proteobacteria were the dominant bacterial phyla, and Ascomycota was the dominant fungal phylum. Genus-level clustering revealed that the bacterial communities under MS and CC were similar to those under CK at the fruiting stage, whereas the fungal communities under all the fumigation treatments were significantly distinct from those under CK. Fumigation effectively inhibited pathogenic genera, including *Amesia*, *Fusarium*, *Rhizopus* and *Ascobolus*, at the seedling recovery stage, but some pathogens recovered at the fruiting stage. The relative abundance of *Fusarium* in the MS treatment increased to 8.25%. DZ treatment performed optimally: it increased beneficial genera such as *Bacillus* and *Streptomyces* at the seedling recovery stage, suppressed harmful genera, including *Amesia* and *Fusarium*, and further enriched *Remersonia* at the fruiting stage. Fumigation significantly improved tomato yield and reduced the incidence of soil-borne diseases. The yield of CC was the highest, at 35.41% greater than that of CK, but it was not significantly different from that of DZ in terms of cost. In conclusion, the DZ treatment had the best overall effect.

## 1. Introduction

Tomato (*Solanum lycopersicum* L.) is an important economic crop in China that is rich in nutrients such as carotene, vitamin C, and lycopene and is widely favored [1,2]. Compared with open-field cultivation, greenhouse cultivation of tomatoes not only significantly increases yield but also promotes early maturation, enabling year-round production and supply [3]. However, facility agriculture commonly faces challenges in terms of crop rotation, leading to the degradation of soil physicochemical properties and the accumulation of soil pathogens, resulting in increasingly severe issues such as continuous cropping obstacles and soil-borne diseases. These problems cause decreases in tomato yield and quality, severely limiting the healthy development of the tomato industry [4,5,6].

The development of soil-borne diseases is closely linked to imbalances in soil microbial communities, often manifested as a significant reduction in beneficial bacterial populations and the enrichment of pathogens. Therefore, reconstructing soil microbial communities and improving the soil microecological balance are crucial for preventing and controlling soil-borne diseases in continuously cropped plants [7,8,9,10]. Currently, in practical production, soil fumigation is the most rapid and effective method for controlling soil-borne diseases. This involves the application of fumigants to sealed soil and the use of volatile active gases to suppress or kill soil-borne pathogens [11]. The acute oral LD50 (rat) values of dazomet, metam sodium, and calcium cyanamide are 320 mg/kg, 450 mg/kg, and 158 mg/kg, respectively (Chemical Toxicity Database https://www.drugfuture.com/toxic/ (accessed on 27 January 2026)), all of which are classified as having low toxicity. Moreover, as small-molecule compounds, they can be rapidly degraded in soil. Therefore, they are the most widely used materials in current production and have been successfully applied for disease control in the production of crops such as tomatoes [12], strawberries [13], potatoes [14], apples [15], and cucumbers [16].

However, evaluations of the effectiveness of different soil fumigants in continuously cropped soils vary, and some scholars have suggested that soil fumigants and their degradation metabolites may have certain negative effects on soil microbial communities and the soil environment, particularly on beneficial microorganisms. After dazomet fumigation, Chen et al. reported that the relative abundance of biocontrol bacteria such as Mortierella in the soil increased, whereas the relative abundance of pathogenic fungi such as Fusarium decreased [17]. Wu et al. reported that after dazomet fumigation, the relative abundance of pathogenic genera such as Ilyonectria and Fusarium in the soil significantly decreased, but the relative abundance of beneficial genera such as Streptomyces, Flavobacterium, Mortierella, and Talaromyces also decreased [18]. Liu et al. reported that calcium cyanamide effectively controlled bacterial wilt and had a relatively small adverse effect on soil microbial activity and community structure [19].

Additionally, fumigation cannot completely eliminate harmful microorganisms in the soil. A certain number of pathogens may survive and proliferate, potentially leading to disease outbreaks [20]. However, there are few reports on the ability of fumigants to alleviate the continuous cropping obstacles associated with protected tomatoes at present. Therefore, this study focused on severely monocropped tomato greenhouses with replanting issues. Three fumigants—dazomet, metham sodium, and calcium cyanamide—were selected. Soil samples were collected during the tomato seedling recovery stage and fruiting period to analyze changes in microbial communities at different postfumigation intervals, as well as direct indicators such as yield and major soil-borne diseases. The aim of this study was to scientifically evaluate the effects of dazomet, metham sodium, and calcium cyanamide fumigation on soil microecosystems and provide theoretical support for the selection of environmentally friendly fumigants.

## 2. Materials and Methods

### 2.1. Experimental Materials

The tested tomato variety was ‘Provence’, a locally predominant cultivar. Dazomet (purity ≥ 98%) was produced by Shizhuang Chemical Co., Ltd., Nantong, China. Metham sodium aqueous solution (purity ≥ 42%) was manufactured by Fengshou Pesticide Co., Ltd., Shenyang, China. Calcium cyanamide (purity ≥ 60%) was obtained from Pengsheng Chemical Co., Ltd., Shizuishan, China.

### 2.2. Experimental Design

The experiments were conducted from 15 July 2024, to 15 February 2025, at Shupeng Farm in Luquan District, Shijiazhuang city, China. Prior to the experiment, the greenhouse had undergone 7 years of continuous tomato cropping, resulting in severe continuous cropping obstacles. The site is located at an altitude of 52 m (35°74′57″ N, 105°36′70″ E), and the basic soil physicochemical properties are as follows: organic matter content of 45.63 g·kg^−1^, total nitrogen content of 2.34 g·kg^−1^, alkali-hydrolyzable nitrogen content of 186.14 mg·kg^−1^, available phosphorus content of 109.98 mg·kg^−1^, available potassium content of 461.23 mg·kg^−1^, and a pH value of 6.17.

Before fumigation, soil samples (5–20 cm topsoil) were collected via a soil auger following the “S”-shaped five-point mixed sampling method. During collection, the top 5 cm of soil was removed; an appropriate amount of soil was taken via the quartering method, placed in a sterile plastic sealable bag, and transported back to the laboratory. After passing through a 2 mm sterile sieve, the soil was aliquoted into cryovials, frozen in liquid nitrogen for 3 h, and then stored at −80 °C for soil microbial diversity analysis. The dosage and application method of the soil fumigants used in the experiments referred to local production practices and previous research by the research group on 30 July 2024, dazomet, metham sodium, and calcium cyanamide were applied uniformly in the experimental plots at rates of 20 kg·667 m^−2^, 40 kg·667 m^−2^, and 80 kg·667 m^−2^, respectively. Three fumigation treatments were implemented, each with three replicates. Each plot covered an area of 50 m^−2^, with a 1 m buffer between plots. The soil was then tilled to a depth of 30 cm via a rotary tiller to ensure thorough mixing of the fumigants with the soil. Subsequently, drip irrigation tubes were laid, and the plots were covered with polyethylene film (thickness ≥ 0.8 mm) to ensure airtightness. After sealing, water was applied via drip irrigation to achieve a soil moisture content of 50–70%. The film remained in place for 30 days. After fumigation, the film was removed, and the soil was aerated for 20 days before healthy tomato seedlings were transplanted. During the seedling recovery period (20 September ), soil samples were collected via the prefumigation method, labeled dazomet fumigation (DZ1), metham sodium fumigation (MS1), and calcium cyanamide fumigation (CC1). Three months after transplanting, when the tomatoes entered the fruiting stage (25 December ), soil samples were collected again and labeled as dazomet fumigation (DZ2), metham sodium fumigation (MS2), and calcium cyanamide fumigation (CC2), with nonfumigated soil serving as the control (CK).

### 2.3. Experimental Methods

Soil genomic DNA was extracted via the Soil DNA Kit produced by Omega Company in the Irving, TX, USA. After quality verification via gel electrophoresis and NanoDrop analysis, high-throughput sequencing of soil fungal ribosomal genes was performed on the Illumina MiSeq platform at Personalbio Technology Co., Ltd. (Shanghai, China). The sequencing region for bacterial ribosomal genes was 16S_V3V4, with the following primer sequences: F: 5′-ACTCCTACGGGAGGCAGCA-3′; R: 5′-GGACTACHVGGGTWTCTAAT-3′. For fungal ribosomal genes, the sequence region was ITS_V1, and the primer sequences were as follows: F: 5′-GGAAGTAAAAGTCGTAACAAGG-3′; R: 5′-GCTGCGTTCTTCATCGATGC-3′. The bacterial 16S rRNA and fungal ITS gene sequences were aligned via the Silva database (https://www.arb-silva.de/ (accessed on 27 January 2026)).

Throughout the growth period of the tomatoes in each fumigation treatment, 5 random sampling points were selected per treatment, with 30 plants per point, to investigate tomato Fusarium wilt, bacterial wilt, and root rot. The incidence rate of each disease was calculated, and the disease index was determined. Incidence rate (%) = (number of infected plants/total number of plants surveyed) × 100; disease index = (Σ(disease severity level × number of plants at that level)/maximum disease severity level × total number of plants) × 100. Within each fumigation treatment plot, 30 tomato plants were randomly selected. The entire harvest process of these plants was recorded, with the yield of each harvest documented (a total of 5 fruit clusters were retained per plant in accordance with local practical production practices). After uprooting, the number of fruits per plant and the single-fruit weight were recorded. Plot yield = average e number of fruits per plant × average single fruit weight × number of plants per plot. Yield (667 m^−2^) = (667 m^−2^/plot area) × plot yield × 85%.

### 2.4. Data Analysis

Paired-end sequencing of the soil community DNA fragments was performed on the Illumina MiSeq platform. The raw sequencing data were stored in FASTQ format. Quality control, denoising, assembly, and chimera removal were conducted via the DADA2 method [21], generating deduplicated sequences after quality control. The microbial community diversity indices (Chao1, observed species, Shannon, and Simpson indices) were calculated with reference to methods described in the literature [22,23]. ASV Venn diagrams, species composition correlation heatmaps, and Spearman correlation heatmaps were generated using the pheatmap package and Venn Diagram package in R language(R 4.4.3). QIIME2 (2019.4) software was used to compare the abundances of taxonomic units at the phylum and genus levels among different treatments and conduct differential analysis. Linear discriminant analysis (LDA) combined with LEfSe (LDA effect size) analysis was employed to perform differential analysis of microbial taxa, thereby identifying differential species in different samples (software: Python LEfSe package in R language(R 4.4.3)). Excel 2021 and SPSS 22.0 were used to conduct significance tests for significant differences between treatments in terms of single-fruit weight, number of fruits per plant, yield, disease incidence, and disease index of tomatoes.

## 3. Results

### 3.1. Effects of Different Fumigant Treatments on Soil Bacterial and Fungal Communities

#### 3.1.1. Soil Sample Depth Evaluation and ASV Clustering Analysis

**Figure 1:**
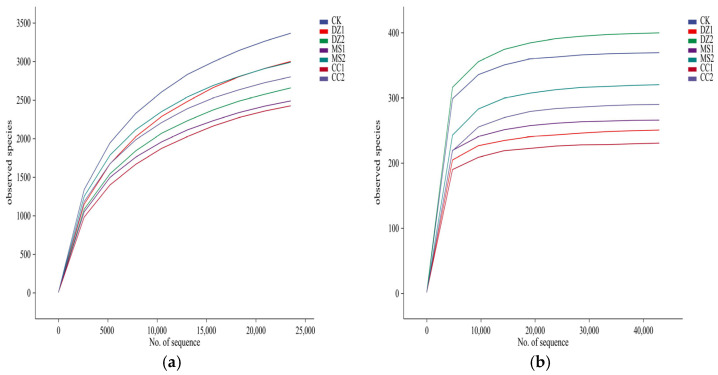
Dilution curves of bacteria (**a**) and fungi (**b**) in soils under different fumigation treatments. Note: DZ1: Soil during the seedling recovery period after dazomet fumigation; DZ2: Soil during the fruiting period after dazomet fumigation; MS1: Soil during the seedling recovery period after metham sodium fumigation; MS2: Soil during the fruiting period after metham sodium fumigation; CC1: Soil during the seedling recovery period after calcium cyanamide fumigation; CC2: Soil during the fruiting period after calcium cyanamide fumigation. The following charts and figures use the same representations.

A total of 1,317,895 pairs of reads were obtained from the bacterial sequences of the 7 soil treatment samples. After clustering at 100% similarity, splicing, and chimera removal, 682,814 high-quality sequences were obtained following paired-end read splicing and filtering, resulting in 46,062 ASVs. Fungal sequencing yielded 1,378,166 pairs of reads. After clustering at 100% similarity, splicing, chimera removal, paired-end read splicing, and filtering, 1,154,809 high-quality sequences were obtained, which were subsequently clustered into 4887 ASVs. Random subsampling of the sequencing sequences was performed, and rarefaction curves were constructed according to the number of sampled sequences and the species they represented. As shown in Figure 1, the rarefaction curves for species numbers gradually flattened, indicating sufficient sequencing depth and reasonable data quantity for subsequent analysis.

**Figure 2:**
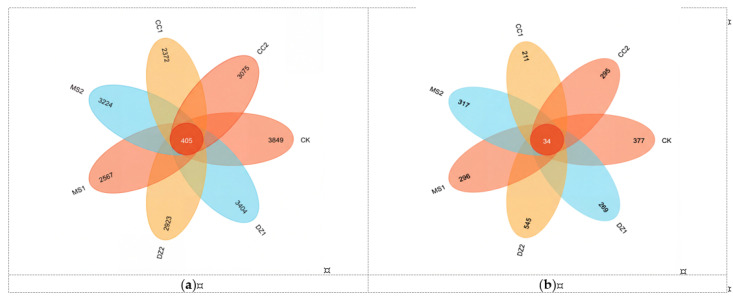
Effects of different fumigation treatments on the distributions of soil bacterial (**a**) and fungal (**b**) ASVs.

As shown in Figure 2a, the 7 treatments shared 405 bacterial ASVs, with the number of unique ASVs in the CK treatment (3849) being the greatest. The unique ASVs for DZ1, MS1, CC1, DZ2, MS2, and CC2 were 11.56%, 24.06%, 33.31%, 16.24%, 24.4%, and 20.11% lower than those of the CK, respectively, with MS2 having the fewest unique ASVs. The data in Figure 2b indicate that the 7 treatments shared 34 fungal ASVs, with DZ2 resulting in the greatest number of unique ASVs, which was 44.56% greater than that in the CK. During the fruiting period, the unique ASV counts for the DZ, MS, and CC treatments were 50.64%, 6.62%, and 28.47% greater, respectively, than those during the seedling acclimation period.

#### 3.1.2. Effects of Different Fumigation Treatments on the α Diversity of Soil Bacteria and Fungi

As shown in Table 1, compared with those in the other treatments, the Chao1 index and observed species index in the CK treatment were significantly greater. The Chao1 index and observed species index of MS2 and CC2 were significantly greater than those of MS1 and CC1. In terms of the bacterial community diversity indices, the Shannon index of the CK treatment was significantly greater than that of the other treatments. The Shannon index and Simpson index of MS2 and CC2 were significantly greater than those of MS1 and CC1. These findings indicate that soil fumigation significantly reduces the richness and diversity of soil bacterial communities. Three months after fumigation, the richness and diversity indices of the bacterial communities in the soils treated with MS and CC increased.

In terms of the fungal community richness indices, compared with those in the CK treatment, the Chao1 index and observed species index in the DZ1, MS1, and CC1 treatments were significantly lower (by 31.53%, 27.96%, and 37.35% and by 32.13%, 27.98%, and 37.55%, respectively). Compared with those of DZ1, MS1, and CC1, the Chao1 index and observed species index of DZ2, MS2, and CC2 significantly increased by 36.88%, 17.07%, and 20.35% and by 37.36%, 16.98%, and 20.5%, respectively. Among the fungal community diversity indices, compared with those in the CK treatment, the Shannon indices in the DZ1, MS1, and CC1 treatments were significantly lower (by 8.7%, 7.75%, and 14.24%, respectively). The Shannon indices of DZ2 and MS2 were significantly greater than those of DZ1 and MS1. The Simpson index of MS2 was significantly greater than that of MS1. These findings indicate that soil fumigation can significantly reduce the richness and diversity of soil fungal communities. Compared with those in the slow seedling stage, the soil fungal community richness indices increased during the fruiting stage in the DZ, MS, and CC treatments, and the soil fungal community diversity indices increased in the DZ and MS treatments.

#### 3.1.3. Analysis of Bacteria and Fungi in the Soil Under Different Fumigation Treatments at the Phylum Level

**Figure 3:**
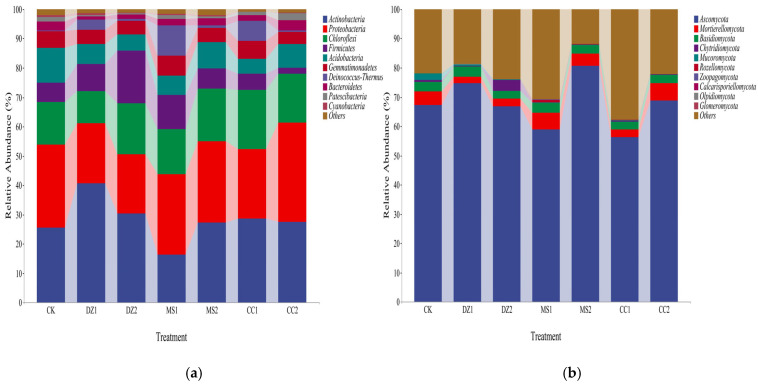
Community structure of soil bacteria (**a**) and fungi (**b**) at the phylum level under different treatments.

As shown in Figure 3a, at the bacterial phylum level, *Actinobacteria* and *Proteobacteria* were the predominant phyla across all the treatments, accounting for 43.7%~61.25% of the total. The relative abundance of *Actinobacteria* was greatest in the DZ1, DZ2, and CC1 treatments, at 40.59%, 30.38%, and 28.65%, respectively. *Proteobacteria* was most abundant in the CK, MS1, MS2, and CC2 treatments (28.28%, 27.42%, 27.69%, and 33.86%, respectively). Compared with that in the CK treatment, the relative abundance of *Actinobacteria* in all the treatments except MS1 was greater, whereas the relative abundance of *Proteobacteria* was lower in all the treatments except CC2 than in the CK. Compared with those in DZ1 and CC1, the abundance of *Actinobacteria* in DZ2 and CC2 decreased by 10.21% and 1.26%, respectively, whereas that in MS2 increased by 10.92% compared with that in MS1. Compared with those in DZ1 and MS1, the relative abundance of *Proteobacteria* in DZ2 and MS2 did not significantly change, whereas that in CC2 increased by 10.24% compared with that in CC1.

As shown in Figure 3b, at the fungal phylum level, *Ascomycota* was the dominant phylum common to all the treatments, accounting for 56.16%~80.61%. Compared with that in the CK treatment, the relative abundance of *Ascomycota* in the DZ1, MS2, and CC2 treatments increased by 7.43%, 13.33%, and 1.58%, respectively, whereas in the DZ2, MS1, and CC1 treatments, it decreased by 0.42%, 8.37%, and 11.11%, respectively. The relative abundance of *Ascomycota* in DZ2 decreased by 7.85 compared with that in DZ1, whereas in MS2 and CC2, the relative abundance of *Ascomycota* increased by 21.7% and 12.68% compared with that in MS1 and CC1, respectively. In summary, after soil fumigation, the relative abundances of bacterial and fungal phyla in the soil changed. Under different fumigation treatments, the trends in relative abundance changes during the recovery and fruiting periods postfumigation exhibited certain differences.

#### 3.1.4. Analysis of the Dominant Bacterial and Fungal Genera in Soil Under Different Fumigation Treatments

A relative abundance > 1% was used as the dominant genus standard. As shown in Table 2, there were 16,17,12,19,16,16 and 18 dominant bacterial genera in CK, DZ1~CC2, respectively. The abundances of the dominant bacterial genera accounted were 32.88%, 44.71%, 42.05%, 39.63%, 33.12%, 41.84% and 35.95%, respectively. Among the seven treatments, six common dominant bacterial communities were identified: *Subgroup_6*, *Micromonospora*, *KD4-96*, *Gitt-GS-136*, *MND1*, and *AKYG1722*. With respect to the dominant fungal genera, the seven treatments (CK, DZ1 to CC2) had 13, 11, 11, 9, 10, and 7 dominant fungal genera, respectively, accounting for 56.71%, 65.59%, 53.43%, 52.53%, 55.44%, 49.17%, and 52.04% of the total, respectively. Among the seven treatments, three common dominant fungal communities were identified: *Aspergillus*, *Mortierella*, and *Remersonia*.

After fumigation, the relative abundance of beneficial and harmful bacterial genera in the dominant flora of each treatment also changed. Two beneficial bacterial genera, *Bacillus* and *Streptomyces*, were detected among the dominant bacterial genera. Compared with that of the control, after the DZ fumigation treatment, the relative abundance of *Bacillus* increased by 1.28% during the seedling recovery stage but further increased to 11.76% at the fruiting stage. Compared with that of the control, the relative abundance of *Streptomyces* increased by 0.53% and 0.73% after the DZ and CC treatments, respectively, during the seedling recovery stage. After the seedling recovery stage, the relative abundance of *Streptomyces* in the DZ treatment increased to 1.58%, whereas that in the CC treatment decreased to 0.95%.

Beneficial fungal genera such as *Aspergillus*, *Remersonia*, *Talaromyces*, *Chaetomium*, *Penicillium*, *Humicola*, and *Trichoderma* were detected among the dominant fungal genera. Compared with those in the CK treatment, the relative abundances of *Remersonia* and *Penicillium* increased to varying degrees after fumigation during the seedling recovery period. Compared with that in the seedling recovery period, the relative abundance of *Penicillium* in the fruiting period decreased, whereas the relative abundance of *Remersonia* significantly increased by 5.07% in the DZ treatment. The relative abundance of *Aspergillus* increased by 10.86% and 10.02% in the DZ and MS fumigation treatments, respectively, during the seedling recovery period but rapidly decreased after the fruiting period began. The relative abundances of *Chaetomium*, *Humicola*, and *Trichoderma* decreased during the seedling recovery period after fumigation, but in the CC treatment during the fruiting period, the relative abundance of Trichoderma increased from 0.25% to 1.2%. Harmful fungal genera such as *Amesia*, *Fusarium*, *Rhizopus*, and *Ascobolus* were detected among the dominant fungal genera. Compared with those of the control, the relative abundances of these four harmful genera decreased during the seedling recovery period after fumigation. *Amesia* decreased by 2.79%, 8.4%, and 10.75% in DZ1, MS1, and CC1, respectively. *Fusarium* decreased by 3.09%, 0.39%, and 3.06% in DZ1, MS1, and CC1, respectively. During the seedling recovery period, the relative abundances of *Rhizopus* and *Ascobolus* in the three fumigation treatments decreased to 0%. Compared with that in the control treatment, the relative abundance of *Amesia* in all the fumigation treatments continued to decrease, with a reduction of 6.24% in the DZ treatment compared with that in the seedling recovery period. However, the relative abundance of *Fusarium* gradually recovered in all the fumigation treatments, although it remained significantly lower than that in the CK in the DZ and CC treatments but increased to 8.25% in the MS treatment. The relative abundance of *Ascobolus* in the CC treatment group recovered to 0.9% during the fruiting period, which was still 0.58% lower than that in the CK group. The results indicate that among the three fumigation treatments, the DZ treatment increased the abundance of beneficial genera while reducing the abundance of most of the pathogenic genera in the soil. Even after three months, which transitioned from the seedling recovery period to the fruiting period, the relative abundance of harmful genera remained suppressed.

**Figure 4:**
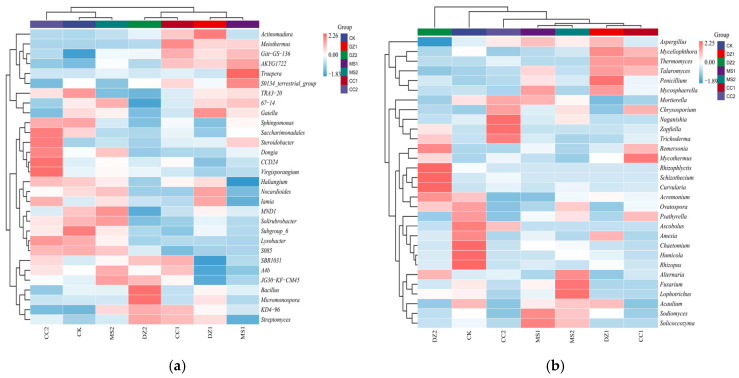
Cluster analysis of soil bacteria (**a**) and fungi (**b**) at the genus level under different treatments.

In terms of the abundance of soil bacteria and fungi, the main bacteria and fungi in the soil of each treatment were concentrated in the top 30 genera. Therefore, a heatmap of the abundance of the top 30 genera was constructed, and a cluster analysis was conducted. The bacterial community composition at the genus level in the different treatments can be divided into three categories: DZ1 and MS1 cluster closely as the first category; DZ2 and CC1 cluster closely as the second category; and MS2, CC2, and CK cluster closely as the third category (Figure 4a). The fungal community composition at the genus level under the different treatments can be divided into five categories: DZ1 and CC1 cluster closely into the first category; MS1 and MS2 cluster closely into the second category; and CC2, CK, and DZ2 cluster in the third to fifth categories (Figure 4b). These results indicate that after the MS and CC treatments, the bacterial community structure gradually approached the prefumigation level during the fruiting stage. The fungal communities in the fumigated treatments significantly differed from those in the CK treatment during both the seedling recovery stage and the fruiting stage. Additionally, the fungal communities in the MS treatments remained similar between the seedling recovery and fruiting stages.

#### 3.1.5. Special Communities of Soil Bacteria and Fungi Under Different Fumigation Treatments

**Figure 5:**
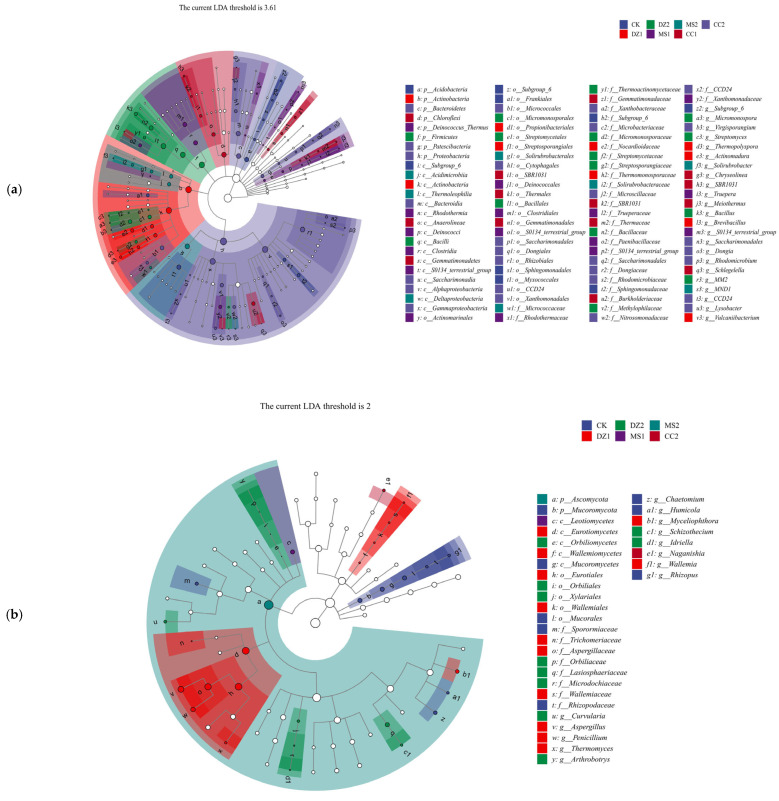
Special communities and influence of bacteria (**a**) and fungi (**b**) in soil after treatment with different soil fumigants. Nodes of different colors represent microbial taxa that are significantly enriched in the corresponding groups and have a significant effect on intergroup differences; lowercase letters represent differential indicator species, where p represents phylum, c represents class, o represents order, f represents family, g represents genus, and s represents species.

LEfSe was used to study the biomarkers with significant differences at different taxonomic levels in the bacterial and fungal communities under different treatments, as shown in Figure 5a, on the basis of the magnitude of community difference, the LDA threshold for the bacterial community was set at 3.61, and a total of 100 differential indicator species were identified, among which 9 were from the CK treatment, including *Acidobacteria*, *Subgroup_6* (from class to genus), *Sphingomonadales* (from order to family), and *Myxococcales.* The DZ treatment resulted in the identification of 10 differential indicator species during the seedling recovery period and 15 during the fruiting period. DZ1 included *Acidobacteria* (from phylum to class), *Propionibacteriales*, *Streptosporangiales*, *Nocardioidaceae*, *Thermomonosporaceae*, *Thermopolyspora*, *Actinomadura*, *Brevibacillus*, and *Vulcaniibacterium*; DZ2 included *Firmicutes*, *Bacilli*, *Micromonosporales*, *Streptomycetales*, *Bacillales*, *Micromonosporaceae*, *Streptomycetaceae*, *Streptosporangiaceae*, *Bacillaceae*, *Methylophilaceae*, *Micromonospora*, *Streptomyces*, *Bacillus*, and *MM2*. The MS treatment resulted in 16 differential indicator species during the seedling recovery period and 8 during the fruiting period. MS1 included *Deinococcus_Thermus*, *Rhodothermia*, *Deinococci*, *Clostridia*, *S0134_terrestrial_group* (from class to genus), *Actinomarinales*, *Deinococcales*, *Clostridiales*, *Rhodothermaceae*, *Trueperaceae*, *Paenibacillaceae*, *Xanthomonadaceae*, and *Truepera*; MS2 included *Acidimicrobiia*, *Thermoleophilia*, *Deltaproteobacteria*, *Solirubrobacterales*, *Micrococcaceae*, *Solirubrobacteraceae*, and *MND1*. The CC treatment resulted in 14 differential indicator species during the seedling recovery period and 28 during the fruiting period. CC1 included *Chloroflexi*, *Anaerolineae*, *Gemmatimonadetes*, *SBR1031* (from order to genus), *Thermales*, *Thermaceae*, *Burkholderiaceae*, *Chryseolinea*, and *Schlegelella*; CC2 included *Bacteroidetes*, *Patescibacteria*, *Proteobacteria*, *Bacteroidia*, *Saccharimonadia*, *Saccharimonadales* (from order to genus), *Dongiales*, *Rhizobiales*, and *CCD24* (from order to genus), and *Xanthomonadales*, *Xanthobacteraceae*, *Microbacteriaceae*, *Microscillaceae*, *Dongiaceae*, *Rhodomicrobiaceae*, *Nitrosomonadaceae*, *Virgisporangium*, *Dongia*, *Rhodomicrobium*, and *Lysobacter*.

As shown in Figure 5b, the fungal community (LDA threshold of 2) produced a total of 33 differential indicator species, among which 8 were differential indicator species for the CK treatment, namely, *Mucoromycota*, *Mucoromycetes*, *Mucorales*, *Sporormiaceae*, *Rhizopodaceae*, *Chaetomium*, *Humicola*, and *Rhizopus*. For the DZ treatment, there were 12 differential indicator species during the seedling recovery stage and 10 during the fruiting stage. DZ1 included *Eurotiomycetes*, *Wallemiomycetes*, *Eurotiales*, *Wallemiales*, *Trichomeriaceae*, *Aspergillaceae*, *Wallemiaceae*, *Aspergillus*, *Penicillium*, *Thermomyces*, *Myceliophthora*, and *Wallemia*; DZ2 included *Orbiliomycetes*, *Orbiliales*, *Xylariales*, *Orbiliaceae*, *Lasiosphaeriaceae*, *Microdochiaceae*, *Curvularia*, *Arthrobotrys*, *Schizothecium*, and *Idriella*. For the MS treatment, there was 1 differential indicator species each during the seedling recovery stage and fruiting stage, namely, *Leotiomycetes* and *Ascomycota*, respectively. For the CC treatment, there was 1 differential indicator species during the fruiting stage, identified as *Naganishia*. The above species influence the structural composition of microbial communities in soils subjected to different fumigation treatments.

### 3.2. Effects of Different Fumigation Treatments on Tomato Yield and Soil-Borne Diseases

As shown in Table 3, the single-fruit weight, number of fruits per plant, and yield were significantly greater in the fumigated treatments than in the CK treatment. Compared with those in the CK treatment, the yields in the DZ, MS, and CC treatments increased by 24.76%, 29.2%, and 35.41%, respectively. Among the soil fumigation treatments, the CC treatment resulted in the highest single-fruit weight (172.2 g), number of fruits per plant (21), and yield (6719.03 kg), but these parameters did not significantly differ between the DZ and MS treatments. As shown in Table 4, no bacterial wilt occurred in any of the fumigated treatments. The incidence and disease indices of Fusarium wilt and root rot in the DZ and CC fumigation treatments were significantly lower than those in the CK, whereas the incidence of Fusarium wilt in the MS treatment was not significantly different from that in the CK. The results indicate that the DZ treatment had the lowest incidence and disease index for soil-borne diseases.

**Figure 6:**
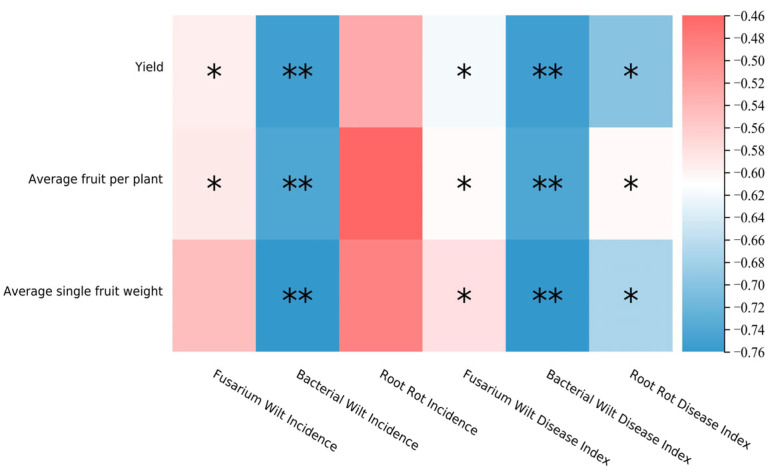
Correlation Heatmap of Yield and Disease Indicators. Note: Darker blue represents a stronger negative correlation and darker red a stronger positive correlation. Asterisks (*) signify significance at *p* < 0.05.

Spearman correlation analysis (Figure 6) revealed that the occurrence of soil-borne diseases had a significant effect on tomato yield. The incidence rate and disease index of bacterial wilt were strongly negatively correlated with yield, number of fruits per plant, and single-fruit weight. The disease index of root rot exhibited a significant weak negative correlation with these yield-related traits. In contrast, the incidence rate and disease index of fusarium wilt were significantly weakly positively correlated with yield, which contradicts the conventional understanding that disease occurrence inhibits yield. A potential explanation for this result is that although both the incidence rate and disease index of soil-borne diseases in the CC fumigation treatment were greater than those in the DZ treatment were, CC, as a slow-release nitrogen fertilizer, could continuously supply nutrients for plant growth. Consequently, the yield of the CC treatment was 528.37 kg per 667 m^2^ greater than that of the DZ treatment.

## 4. Discussion

The occurrence and exacerbation of crop continuous cropping obstacles are related to the combined effects of multiple factors within the plant–soil–microbial system. Among these factors, the imbalance of soil microecology is a significant cause of frequent soil-borne diseases [24]. An imbalance in soil microecology is manifested mainly by a reduction in beneficial microorganisms, enrichment of pathogenic bacteria, and transformation of microbial community characteristics from “bacterial-type” to “fungal-type” [25]. Soil fumigation can eliminate target microorganisms in the soil, reconstruct soil microbial community structures, effectively control soil-borne diseases in crops, and alleviate continuous cropping obstacles [26]. Therefore, evaluating and clarifying the impact and persistence of different fumigants on soil microbial communities are important for the prevention and control of continuous cropping obstacles.

In this study, three fumigants—dazomet, metham sodium, and calcium cyanamide—were selected. Soil samples were collected during the tomato seedling recovery period and fruiting period to analyze changes in microbial diversity and community structure after fumigation. After fumigation, the richness and diversity of the soil bacterial and fungal communities decreased to varying degrees, which is consistent with the findings of Chen et al. [27]. Compared with those during the seedling recovery period, the soil fungal community richness index increased during the fruiting period, and the richness and diversity indices of the soil bacterial communities in the MS and CC treatments increased. These findings indicate that three months after fumigation, the soil microbial diversity gradually recovered, which aligns with the results of De et al. [20].

The soil in continuously cropped fields contains many pathogenic bacteria, and soil fumigation can eliminate most of them. After fumigation, the relative abundances of *Amesia*, *Fusarium*, *Rhizopus*, and *Ascobolus* decreased during the seedling recovery period. *Amesia* can cause root rot, stem rot, and leaf spot symptoms in plants [28]. *Fusarium* is a common plant pathogen, and multiple species within this genus are closely related to soil-borne diseases in crops [29,30]. For example, wilt pathogens (*Fusarium oxysporum*) can invade the young roots or wounds of tomato plants, leading to yellowing and wilting of leaves. This pathogen has a wide host range and is infectious [31]. *Fusarium graminearum* causes root rot, and once the disease occurs, it is difficult to eliminate in the short term [32]. *Rhizopus* can cause root rot in plants [33]. *Ascobolus* accumulates in the soil over time and is strongly positively correlated with disease incidence [34]. However, after the fruiting period, the abundance of *Amesia* continued to decline in the DZ treatment, with a decrease of 6.24% compared with that in the seedling recovery period, whereas the relative abundance of *Fusarium* gradually recovered in all the fumigation treatments. Nevertheless, compared with those in the CK treatment, the relative abundances in the DZ and CC treatments remained significantly lower, whereas that in the MS treatment increased to 8.25%. The results of these experiments revealed that fumigation can effectively control *Fusarium* species, but the inhibition rate of pathogens is greater during the seedling recovery period. This finding is also consistent with the findings of this study, which revealed that fumigation significantly reduced the incidence and disease indices of wilt and root rot.

Soil microorganisms are key for maintaining the stability of soil ecosystems, and an increase in the number of beneficial microorganisms is crucial for maintaining the dynamic balance of soil microecology. However, the broad-spectrum nature of fumigation leads to the suppression of some beneficial microbial communities while eliminating pathogenic bacteria. However, some studies have shown that the relative abundance of certain soil microbial communities does not decrease after soil fumigation but instead increases [35]. Compared with those in the CK treatment, the relative abundances of *Remersonia* and *Penicillium* increased to varying degrees during the seedling recovery period after the fumigation treatment. Compared with that in the seedling recovery period, the relative abundance of *Penicillium* in the fruiting period decreased, whereas the relative abundance of *Remersonia* significantly increased by 5.07% in the DZ treatment. Remersonia can promote soil nutrient absorption and inhibit the development of pathogenic bacteria in the soil [36]. *Penicillium* can secrete broad-spectrum antimicrobial compounds and has the potential to promote plant growth [37,38]. The relative abundance of *Aspergillus* increased by 10.86% and 10.02% in the DZ and MS fumigation treatments, respectively, during the seedling recovery period but rapidly decreased after the fruiting period began. After fumigation, the relative abundances of *Chaetomium*, *Humicola*, and *Trichoderma* decreased during the seedling recovery period. However, in the CC treatment during the fruiting period, the relative abundance of *Trichoderma* increased from 0.25% to 1.2%. After the DZ fumigation treatment, the relative abundance of *Bacillus* increased by 1.28% during the seedling recovery period but further increased to 11.76% upon entering the fruiting period. The relative abundance of *Streptomyces* increased by 0.53% and 0.73% in the DZ and CC treatments, respectively, during the seedling recovery period. After the seedling recovery period, the relative abundance of *Streptomyces* in the DZ treatment increased to 1.58%, whereas that in the CC treatment decreased to 0.95%. *Aspergillus* can purify soil contaminated by microbial toxins [39]. *Chaetomium* has been reported to act as a broad-spectrum fungicide for controlling plant diseases [40]. *Humicola* can reduce the incidence of wilt and black spot diseases [41,42]. *Talaromyces* can inhibit various pathogenic microorganisms [43,44]. *Trichoderma* is widely used as a plant growth promoter and biocontrol agent in crop production [45]. *Bacillus* can secrete antimicrobial peptides to suppress soil-borne pathogens, such as those causing wilt and bacterial wilt [46]. *Streptomyces* can secrete multiple antibiotics and has significant effects on the prevention and control of tomato bacterial wilt and Fusarium wilt [47].

Through genus-level cluster analysis, this study revealed that the bacterial communities treated with MS and CC gradually approached prefumigation levels after they entered the fruiting stage. The fungal communities in the fumigation treatments significantly differed from those in the CK treatment during both the seedling recovery stage and the fruiting stage. Therefore, the three fumigation treatments (DZ, MS, and CC) effectively eliminated most of the pathogenic bacterial genera in the soil during the seedling recovery stage. However, by the fruiting stage, only the DZ treatment continued to suppress the relative abundance of harmful bacterial genera while increasing the abundance of some beneficial genera. The enrichment of these beneficial genera is important for reducing soil pathogen abundance, inhibiting population rebound, and maintaining a stable and healthy soil microecosystem. Among the three fumigation treatments, the DZ treatment presented the lowest incidence rate and disease index of soil-borne diseases. However, its yield was 528.37 kg lower than that of the CC treatment, which contradicts the conventional understanding that disease suppression improves yield. Spearman correlation analysis revealed that the disease indicators of bacterial wilt and root rot were negatively correlated with yield indicators, whereas the incidence rate and disease index of fusarium wilt were significantly weakly positively correlated with yield. As a slow-release nitrogen fertilizer, CC has been proven by previous studies to continuously provide nutrients for plant growth, thereby increasing crop yield, which may be the reason for this abnormal phenomenon [48].

## 5. Conclusions

Soil fumigant application significantly reduced the richness and diversity of the bacterial and fungal communities in continuously cropped tomato soil during the seedling recovery stage. Three months after fumigation, the soil microbial diversity gradually recovered. A comprehensive analysis of the dominant bacterial and fungal genera revealed that among the three fumigation treatments, the DZ treatment increased the abundance of beneficial bacterial genera while reducing the abundance of the vast majority of pathogenic genera in the soil. After three months, when the seedling recovery stage transitioned to the fruiting stage, the relative abundance of harmful genera remained inhibited. Compared with the DZ treatment, the CC treatment resulted in the highest yield index, but the difference was not significant; in addition, the cost of CC was relatively high. Therefore, the DZ treatment resulted in optimal comprehensive performance and promoted tomato production.

Under the conditions of this experiment, although the DZ treatment performed the best, considering the broad-spectrum killing effect of fumigants on soil microorganisms and the timeliness of their inhibitory effect on pathogenic communities, supplementation with beneficial microorganisms (such as microbial fertilizers) after fumigation is necessary. In addition, this study monitored only the impact of fumigants on microbial communities during the growth period, did not verify long-term soil health indicators such as soil enzyme activity and nutrients, and ignored factors such as fumigant residues and climate fluctuations in actual production. In future research, continuous field experiments should be carried out to detect the dynamic changes in fumigant residues, soil enzyme activity, nutrients and other indicators. Moreover, multiple environmental factor experiments should be performed to fully evaluate and screen environmentally friendly fumigants.

## Figures and Tables

**Table 1:** Soil bacterial and fungal community richness and diversity indices under different fumigation treatments.

Kingdom	Treatment	Community Abundance Index		Community Diversity Index	
		Chao1 Index	Observed Species	Shannon	Simpson
Bacteria	CK	3882.77 ± 211.97 a	3366.47 ± 88.34 a	10.73 ± 0.05 a	0.9989 ± 0.0001 a
	DZ1	3466.94 ± 134.27 b	3001.10 ± 162.29 b	10.00 ±0.15 c	0.9961 ± 0.0008 bc
	DZ2	3019.60 ± 98.12 cd	2653.87 ± 51.71 cd	9.88 ± 0.12 cd	0.9964 ± 0.0007 b
	MS1	2892.61 ± 259.11 d	2488.50 ± 168.63 d	9.73 ± 0.24 de	0.9949 ± 0.0017 c
	MS2	3354.01 ± 134.27 bc	2988.30 ± 65.43 b	10.49 ± 0.04 ab	0.9986 ± 0.0001 a
	CC1	2845.14 ± 275.04 d	2426.53 ± 226.33 d	9.50 ±0.16 e	0.9950 ± 0.0007 bc
	CC2	3105.69 ± 215.78 cd	2801.47 ± 132.86 bc	10.29 ± 0.07 b	0.9982 ± 0.0001 a
Fungi	CK	369.17 ± 16.94 a	368.83 ± 17.02 a	6.32 ± 0.10 ab	0.9676 ± 0.0051 a
	DZ1	252.78 ± 36.41 cd	250.33 ± 33.30 cd	5.77 ± 0.14 cd	0.9577 ± 0.0091 ab
	DZ2	400.45 ±22.41 a	399.63 ± 21.60 a	6.62 ± 0.46 a	0.9745 ± 0.0082 a
	MS1	265.94 ±11.48 cd	265.63 ± 11.47 cd	5.83 ± 0.16 bc	0.9571 ± 0.0079 ab
	MS2	320.68 ± 40.62 b	319.97 ± 40.94 b	5.22 ± 0.33 e	0.9202 ± 0.0222 c
	CC1	231.30 ± 19.48 d	230.33 ± 19.16 d	5.42 ± 0.23 cde	0.9403 ± 0.0064 bc
	CC2	290.40 ± 9.46 bc	289.73 ± 9.48 bc	5.28 ± 0.37 de	0.9324 ± 0.0209 c

Note: Significant (*p* < 0.05) differences are expressed with different letters.

**Table 2:** Changes in the relative abundances of the dominant bacterial and fungal genera in the soil under the different fumigation treatments.

Kingdom	Serial Number	Genus	CK	DZ1	DZ2	MS1	MS2	CC1	CC2
			%						
Bacteria	1	*Subgroup_6*	8.10	5.01	4.19	4.23	5.72	3.74	5.72
	2	*Bacillus*	3.48	4.76	11.76	3.29	3.41	2.27	0.89
	3	*SBR1031*	3.04	0.99	4.95	2.58	3.77	5.33	4.44
	4	*Actinomadura*	0.26	11.08	2.31	1.02	0.91	6.57	0.48
	5	*Micromonospora*	1.88	3.82	7.63	1.29	1.91	1.58	1.73
	6	*A4b*	2.01	0.84	2.12	1.26	2.95	2.73	2.35
	7	*KD4-96*	1.53	1.91	2.22	1.77	2.03	2.15	1.55
	8	*Meiothermus*	0.00	3.32	0.28	3.43	0.00	6.05	0.01
	9	*Gitt-GS-136*	1.40	1.91	1.77	2.04	1.78	2.09	1.65
	10	*MND1*	1.91	1.70	1.17	1.54	2.14	1.48	1.54
	11	*AKYG1722*	1.11	1.67	1.26	1.90	1.46	1.75	1.17
	12	*Truepera*	0.25	0.28	0.38	6.90	0.82	0.70	0.29
	13	*CCD24*	1.11	0.83	1.09	1.03	1.23	1.24	1.95
	14	*Sphingomonas*	1.64	0.77	0.76	1.21	1.03	1.12	1.60
	15	*Streptomyces*	0.73	1.26	1.58	0.50	0.90	1.46	0.95
	16	*Saccharimonadales*	1.33	0.41	0.54	0.95	0.59	0.85	2.07
	17	*S0134_terrestrial_group*	0.95	0.92	0.97	1.57	0.59	1.16	0.54
	18	*Steroidobacter*	0.71	0.88	0.70	1.17	0.80	0.87	1.54
	19	*Haliangium*	1.07	1.02	0.75	0.55	0.98	0.94	1.11
	20	*Virgisporangium*	0.73	0.19	0.75	0.37	1.01	0.53	2.80
	21	*67-14*	0.96	1.02	0.54	1.09	1.16	0.82	0.71
	22	*Gaiella*	0.98	1.19	0.66	0.95	0.92	0.80	0.67
	23	*Nocardioides*	1.06	1.43	0.47	0.33	1.23	0.47	0.87
	24	*Dongia*	0.69	0.45	0.38	0.64	1.03	0.45	1.38
	25	*Solirubrobacter*	1.12	0.61	0.26	0.43	1.26	0.36	0.85
	26	*Lysobacter*	1.08	0.37	0.33	0.35	0.71	0.38	1.21
	27	*Brevibacillus*	0.06	1.19	0.87	1.02	0.02	0.85	0.00
	28	*Acidibacter*	0.46	0.30	0.63	0.53	0.52	0.47	1.07
	29	*Vulcaniibacterium*	0.00	1.27	0.03	0.94	0.0	1.11	0.00
	30	*Paenisporosarcina*	0.19	0.17	0.17	1.28	0.68	0.09	0.33
	31	*Thermopolyspora*	0.00	1.17	0.51	0.09	0.00	0.80	0.00
	32	*Rhodomicrobium*	0.26	0.10	0.19	0.18	0.41	0.24	1.07
	The relative abundance > 1% of the genus number.		16	17	12	19	16	16	18
	Dominant genus proportion/%		32.88	44.71	42.05	39.63	33.12	41.84	35.95
Fungi	1	*Aspergillus*	15.67	26.53	2.89	25.69	20.14	15.05	20.55
	2	*Alternaria*	3.70	0.59	10.78	1.69	13.56	3.38	4.16
	3	*Mortierella*	4.65	2.16	2.67	5.75	4.24	2.78	5.96
	4	*Zopfiella*	0.00	0.00	5.99	0.00	0.00	1.23	17.65
	5	*Myceliophthora*	3.54	9.16	0.87	1.16	1.96	7.01	0.29
	6	*Amesia*	10.75	7.96	1.72	2.35	0.30	0.00	0.00
	7	*Remersonia*	1.39	2.39	7.46	2.87	1.28	5.70	1.41
	8	*Fusarium*	3.30	0.21	1.15	2.91	8.25	0.24	0.69
	9	*Talaromyces*	0.57	4.93	0.18	3.33	1.06	4.10	0.92
	10	*Acremonium*	3.15	2.25	4.01	0.85	2.01	1.96	0.78
	11	*Sodiomyces*	1.75	1.68	0.82	4.26	2.94	0.38	0.11
	12	*Thermomyces*	0.04	4.52	0.08	0.00	0.00	4.48	0.87
	13	*Schizothecium*	0.00	0.00	8.82	0.00	0.00	0.00	0.96
	14	*Mycothermus*	0.25	1.15	1.82	0.49	0.07	3.50	0.96
	15	*Chaetomium*	3.16	0.17	0.25	0.76	0.65	0.05	0.15
	16	*Rhizophlyctis*	0.51	0.00	3.56	0.00	0.00	0.00	0.00
	17	*Penicillium*	0.05	1.62	0.29	0.74	0.30	0.41	0.14
	18	*Humicola*	2.24	0.00	0.00	0.00	0.00	0.00	0.01
	19	*Mycosphaerella*	0.2	1.24	0.1	1.23	0.03	0.3	0.03
	20	*Rhizopus*	1.92	0.00	0.00	0.00	0.00	0.00	0.00
	21	*Trichoderma*	0.12	0.06	0.70	0.20	0.02	0.25	1.20
	22	*Solicoccozyma*	0.28	0.00	0.07	1.30	0.84	0.00	0.00
	23	*Ascobolus*	1.48	0.00	0.00	0.00	0.00	0.00	0.9
	24	*Curvularia*	0.00	0.04	1.44	0.00	0.16	0.06	0.14
	25	*Naganishia*	0.12	0.00	0.00	0.09	0.36	0.00	1.11
	26	*Idriella*	0.00	0.00	1.11	0.00	0.05	0.00	0.00
	The relative abundance > 1% of the genus number.		13	11	13	11	9	10	7
	Dominant genus proportion/%		56.71	65.59	53.43	52.53	55.44	49.17	52.04

**Table 3:** Effects of different fumigation treatments on the yield of tomato.

Treatment	Average Fruit Per Plant (Units)	Average Single Fruit Weight (g^−1^)	Yield kg·667 m^−2^
CK	18.00 ± 1.00 b	145.67 ± 6.6 b	4962.08 ± 119.40 b
DZ	20.33 ± 1.53 a	163.8 ± 3.72 a	6190.66 ± 428.39 a
MS	20.67 ± 0.58 a	166.57 ± 3.45 a	6402.03 ± 247.83 a
CC	21.00 ± 1.00 a	172.2 ± 7.65 a	6719.03 ± 280.19 a

Note: Significant (*p* < 0.05) differences are expressed with different letters.

**Table 4:** Effects of Different Fumigation Treatments on Tomato Soil-borne Diseases.

Treatment	Incidence %			Disease Index		
	Fusarium Wilt	Bacterial Wilt	Root Rot	Fusarium Wilt	Bacterial Wilt	Root Rot
CK	17.33 ± 2.67 a	4.67 ± 1.70 a	15.33 ± 1.70 a	9.60 ± 2.14 a	4.60 ± 2.04 a	12.60 ± 1.54 a
DZ	4.00 ± 1.94 b	0.00 ± 0.00 b	2.67 ± 1.24 c	1.60 ± 0.86 b	0.00 ± 0.00 b	0.80 ± 0.39 c
MS	12.67 ± 2.86 a	0.00 ± 0.00 b	8.67 ± 1.33 b	6.00 ± 1.67 ab	0.00 ± 0.00 b	4.53 ± 1.85 b
CC	5.33 ± 1.70 b	0.00 ± 0.00 b	4.67 ± 1.33 bc	2.4 ± 0.78 b	0.00 ± 0.00 b	1.47 ± 0.68 c

Note: Significant (*p* < 0.05) differences are expressed with different letters.

## Data Availability

The manuscript contains all data that were created or examined during the research.
